# The Mechanism of Budding of Retroviruses from Cell Membranes

**DOI:** 10.1155/2009/623969

**Published:** 2009-03-05

**Authors:** Andrew Pincetic, Jonathan Leis

**Affiliations:** Department of Microbiology and Immunology, Feinberg School of Medicine, Northwestern University, Chicago, IL 60611, USA

## Abstract

Retroviruses have evolved a mechanism for the release of particles from the cell membrane that appropriates cellular protein complexes, referred to as ESCRT-I, -II, -III, normally involved in the biogenesis of multivesicular bodies. Three different classes of late assembly (L) domains encoded in Gag, with core sequences of PPXY, PTAP, and YPXL, recruit different components of the ESCRT machinery to form a budding complex for virus release. Here, we highlight recent progress in identifying the role of different ESCRT complexes in facilitating budding, ubiquitination, and membrane targeting of avian sarcoma and leukosis virus (ASLV) and human immunodeficiency virus, type 1 (HIV-1). These findings show that retroviruses may adopt parallel budding pathways by recruiting different host factors from common cellular machinery for particle release.

## 1. Introduction

Due to the small size and content of their genomes, retroviruses rely on host cell as much as viral encoded enzymes, for successful replication. This is particularly evident in one of the least understood aspects of the retrovirus life cycle: the assembly and release of virus particles from the cell surface. When expressed in the absence of other viral components, Gag (encoding the structural proteins of the virus) forms virus-like particles (VLPs) that bud from cells, independent of an active viral-encoded protease (PR). The assembly process is driven primarily by elements within Gag [1], such as the membrane-targeting (M) domain, the Gag-Gag interaction (I) domain necessary for particle formation, and the late assembly (L) domain required for the separation of the virion from the host cell membrane. The L domain recruits an ATP-requiring cellular factor for this scission event, the only known energy-dependent step in assembly [2]. Domain is used here to denote the amino acid sequence that constitutes the biological function.

The first suggestions of a virally encoded element responsible for particle budding came from studies of HIV-1 mutants in which stop codon mutations introduced into the p6 region of Gag-blocked virion release [3]. The function of the L domain was subsequently defined in both ASLV and HIV-1, and mapped to proline-rich sequences in the p2b [4, 5] and p6 [6] region of Gag, respectively. Single amino acid substitutions in the PPPY sequence in ASLV Gag or the PTAP sequence in HIV-1 Gag caused fully assembled virus particles to accumulate on the cell surface tethered by a thin membrane stalk. There are now three distinct L domains identified and associated with budding defects within the retrovirus family, with core amino acid motifs of PPxY, P(T/S)AP, and YPxL [7]. Interestingly, the same L domain motifs are found in other enveloped virus families (such as filoviruses, rhabdoviruses, and arenaviruses) [8]. These similarities suggest that simple enveloped viruses may have evolved related budding mechanisms for mediating their release from cell membranes.

L domains share a set of characteristics that help define their function. First, they exhibit positional independence in that the peptide motif can be shifted to different regions within Gag without disrupting budding [5, 9]. Second, they are functionally exchangeable in that the L domain from one retrovirus can substitute that of another [10]. In some cases, the L domain can function in the context of different virus families [11]. Third, L domains exert their function in the final stages of virion assembly as evidenced by the delay in proteolytic processing of Gag-bearing L domain substitutions [6, 12]. These sequences can thus be defined as protein-binding modules that recruit the host cell factors that mediate the release of tethered virus particles from the cell membrane. Though a single L domain is sufficient for particle release for many viruses (i.e., ASLV or equine infectious anemia virus (EIAV)), L domain motifs frequently appear in combinations. For example, the Mason-Pfizer monkey virus (M-PMV) and human T-cell leukemia virus (HTLV-1) contain tandem PPxY and PTAP motifs. HIV-1 Gag contains a secondary YPX(n)L-type motif downstream of the PTAP sequence. In the context of multiple L domains, one motif usually serves a dominant role in budding. This means that the substitution of the dominant L domain has a strong effect on blocking budding while the substitution of the auxiliary L domain has smaller inhibitory effect in comparison. The PPPY motif is dominant over the PS/TAP motif in M-PMV [13] and HTLV-1 Gag [14], and the PTAP motif is dominant over the YPLTSL motif in HIV-1 Gag [15]. These observations suggest that different types of L domains may dictate different budding pathways, and the presence of multiple L domain motifs within Gag may provide redundant or synergistic properties for efficient particle release in the various cell types infected in vivo. 

Many studies have focused on identifying the host cell factors recruited by each L domain. For HIV-1 Gag, the dominant PTAP motif binds to Tsg101 [16], and the secondary YPLTSL motif binds weakly to AIP1/Alix [15, 17, 18]. The YPDL motif of EIAV Gag also binds to AIP1/Alix [19], though with much greater affinity than HIV-1 Gag [19, 20]. The PPPY motif of ASLV Gag and murine leukemia virus (MLV) Gag binds to members of the Nedd4 family of E3 ubiquitin ligases [21, 22]. Tsg101 and AIP1/Alix belong to the class E Vps protein family which functions in the biogenesis of multivesicular bodies (MVBs) in eukaryotic cells. MVBs are intermediate endosomes that transport cargo proteins from early to late endosomes for subsequent degradation in the lysosome. Cargo proteins designated for degradation are sorted into luminal vesicles of MVBs by class E Vps proteins in a process topologically equivalent to virus budding [23]. Genetic screens of *Saccharomyces cerevisiae * originally identified ~18 class E Vps proteins, most of which are assembled into three high-molecular weight cytoplasmic complexes called endosomal sorting complexes required for transport (ESCRT)-I, -II, and -III. Alignments with yeast coding sequences revealed that mammalian cells contain ~30 orthologues of the class E Vps proteins implicating a highly conserved MVB pathway for sorting cargo proteins in eukaryotic cells. Like virus budding, vesicle formation requires ATP hydrolysis to break attachment to cellular membranes. The ESCRT-III complex recruits the AAA ATPase, Vps4, to endosomal membranes to facilitate membrane scission and recycle membrane-bound ESCRT complexes to the cytosol [24, 25]. The same Vps4 protein is required by retroviruses to bud because the coexpression of a catalytically inactive form of Vps4, Vps4_E228Q_, arrests particles at the plasma membrane [26, 27]. These findings validate the conclusion that retroviruses, regardless of which L domain they encode, rely on the ESCRT machinery for budding. 

## 2. Budding Complexes

**Figure 1:**
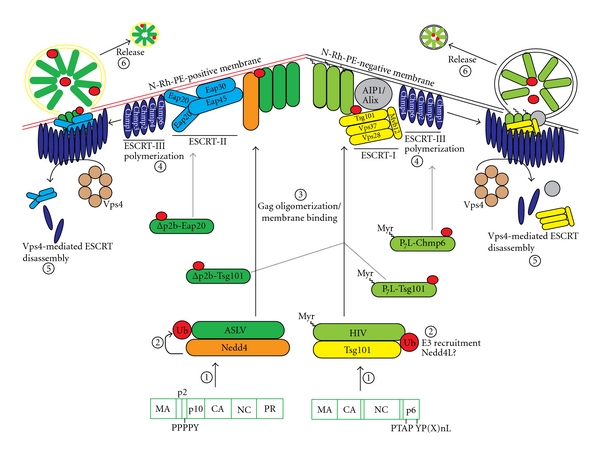
Parallel pathways in ASV and HIV-1 Gag budding. Retroviruses recruit components of the ESCRT machinery to build a budding complex for particle release. (1) The dominant L domains for HIV-1 and ASV Gag bind to Tsg101 and Nedd4, respectively. Whether this initial interaction takes place in the cytosol or at the plasma membrane remains to be defined. (2) Nedd4 mediates ubiquitination of ASV Gag. HIV-1 Gag is ubiquitinated by an unidentified E3 ligase. Some evidence suggests that Nedd4L may play a role since its overexpression rescues budding of HIV-1 Gag/ΔPTAP. (3) Gag oligomerization in the cytosol increases membrane avidity and rapidly targets Gag to sites of assembly/budding on the plasma membrane. ASV Gag assembles on N-Rh-PE-positive, endosome-derived membranes. HIV-1 Gag assembles on N-Rh-PE-negative membranes. (4) During the budding process, Gag may recruit additional ESCRT factors eventually leading to ESCRT-III polymerization at the base of a budding particle. (5) ESCRT-III subunits recruit the AAA ATPase, Vps4, to mediate the disassembly of membrane-bound ESCRT complexes and to provide the energy for membrane fission. (6) VLPs are released from cellular membranes. Covalently linking ESCRT proteins to the C-terminus of Gag bearing L domain-mutations restores budding at different stages. Tethering Tsg101 to ASV Gag/Δp2b (Δp2b-Tsg101) or HIV-1 Gag/P7L (P7L-Tsg101) rescues budding through an HIV-like pathway (ESCRT-I-dependent, N-Rh-PE-negative membranes). Tethering EAP20 to ASLV Gag/Δp2b (Δp2b-Eap20) rescues budding through an ASLV-like pathway (ESCRT-II-dependent, N-Rh-PE-positive membranes). Black arrows indicate wild-type Gag budding pathways. Gray arrows indicate reconstituted budding pathways of ASLV Gag/Δp2b-ESCRT fusions and HIV-1 Gag/P7L-ESCRT fusions.

The finding that retroviruses bind to components of a very specialized protein transport/membrane fission network suggests that L domains must recruit a minimal set of host cell proteins to form a “budding complex” for particle release. Because retroviruses encode different L domains that directly bind different cellular factors, the constituents that make up the budding complex for each retrovirus may differ.  Figure 1 summarizes the differences and similarities of the ASLV and HIV-1 budding pathways. When L domains are exchanged between retroviruses, the heterologous L domain confers different budding properties on Gag. For example, the release of wild-type EIAV Gag is resistant to dominant-negative inhibition by the C-terminal fragment of Tsg101 (Tsg-3′) and proteasome inhibitors, which normally inhibits both HIV-1 Gag and MLV Gag. However, replacing the YPDL L domain of EIAV Gag with the PTAP L domain of HIV-1 Gag or the PPPY L domain of MLV Gag renders EIAV sensitive to both Tsg-3′ and inhibitor treatment [28]. Presumably, the differences in budding properties with heterologous L domains reflect the reconstitution of a parallel budding pathway in which different host complexes are utilized for budding. 

The PPPY motif of ASLV Gag binds to a Nedd4-like E3 ligase to facilitate budding (see Figure 1) [21, 29]. There are ~10 known members of the Nedd4 family and they form a subset of a larger contingent of as many as 1500 E3 proteins. The interaction of Nedd4-like proteins with Gag is mediated by WW motifs which form protein interaction modules that bind proline-rich sequences 11–14 amino acids in length. Additionally, Nedd4-like proteins also contain a C-terminal HECT domain required for the transfer of ubiquitin to the target protein, and, in most splicing variants, an N-terminal C2, Ca^+2^-dependent transport domain that targets E3 proteins to the cell membrane. Coexpression of an avian Nedd4-like E3 protein made catalytically inactive by a single amino acid substitution in its HECT domain inhibits release of ASLV Gag, providing strong evidence that ubiquitin signaling plays an important role in VLP release [29]. Yet, translational fusion of ubiquitin to ASLV Gag failed to rescue particle release in the presence of the dominant negative avian Nedd4-like fragment called LDI-1 (C2-WW). This suggests that a full-length Nedd4-like protein (C2-WW-HECT) contributes in addition critical functions during release besides the ubiquitination of Gag [29]. One possibility is that Nedd4-like E3 proteins function as adaptors to link ASLV Gag to ESCRT components of the endocytic pathway. Unlike Tsg101 or AIP1, Nedd4-like proteins are not known to function in MVB biogenesis in mammalian cells. However, several lines of evidence suggest that ASLV Gag, like HIV-1 Gag, relies on the ESCRT machinery for budding. First, expressing the catalytically inactive Vps4_E228Q_ enzyme inhibits ASLV Gag release in a dominant-negative fashion [26]. Second, it was previously established that Nedd4 interacts with Tsg101 [26]. The relevance of this interaction is supported by the observation that overexpressing Nedd4L rescues the budding defect caused by the PTAP deletion in HIV-1 Gag [12, 30] and that this Nedd4L-mediated rescue required Tsg101. However, unlike the budding pathway of wild-type HIV-1 Gag, Nedd4L-mediated rescue of HIV-1 ΔPTAP release did not require the PTAP or the ubiquitin-binding ability of Tsg101 [12, 30]. Third, Tsg101 can substitute for the L domain function of ASLV Gag in promoting efficient VLP release [31]. Nevertheless, Tsg101 does not normally appear to play a role in ASLV Gag budding because Tsg101 depletion in mammalian and/or avian cells fails to block VLP release [31]. Finally, multiple Nedd4 family members localize to abnormal endosomes, called class E compartments, formed in the presence of Vps4_E228Q_ [22]. These compartments sequester ESCRT complexes and other proteins associated with the ESCRT machinery on the limiting endosomal membrane. Despite these three lines of evidence, the mechanism by which Nedd4-like proteins link ASLV Gag to downstream ESCRT factors is not yet understood.

Because L domain motifs link Gag to the ESCRT machinery, ESCRT proteins can functionally substitute for these motifs when covalently linked to the C-terminus of Gag carrying L domain-inactivating substitutions. For example, Tsg101 rescues HIV-1 Gag budding when tethered to the C-terminus of HIV-1 Gag/Δp6 (Figure 1) [32]. Constructing such Gag-ESCRT chimeras provides a valuable complementation assay to examine the role of ESCRT proteins in retrovirus egress. Vps37B [33] and Vps37C [34] also rescue budding of HIV-1 Gag/P_7_L (an inactivating PTAP substitution) confirming the view that HIV-1 requires ESCRT-I activity for release. When proteins from the ESCRT-II and -III complexes are fused to HIV-1 Gag/P_7_L, only the ESCRT-III protein, Chmp6, restored efficient VLP release (see Figure 1) [34]; the ESCRT-II proteins, Eap20 and Eap45, failed to rescue particle release which correlates with previous reports that HIV-1 Gag does not require ESCRT-II proteins [35]. In contrast, Eap20 partially complements the Δp2b deletion when conjugated to the C-terminus of ASLV Gag/Δp2b. This suggests a functional requirement for ESCRT-II in ASLV Gag budding (see Figure 1). In support of this view, siRNA-mediated depletion of Eap20, which does not interfere with HIV-1 Gag budding, potently inhibits the release of ASLV Gag VLPs [34]. This is the first demonstration of the ESCRT-II complex participating in retrovirus budding. The finding that HIV-1 and ASLV Gag differ in their requirement for ESCRT-I and -II complexes supports the hypothesis that different L domains specify the use of different ESCRT complexes during budding.

Retroviruses that are sensitive to dominant-negative Vps4 most likely share a common requirement for ESCRT-III. The ESCRT-III complex consists of ~11 CHMP proteins characterized as small, highly-charged coiled-coil-containing proteins that oligomerize into an array on endosomal membranes. Charged MVB proteins (CHMPs) contain a highly basic N-terminus and an acidic C-terminus, which allows these proteins to adopt either an “open” or “closed” conformation [36]. In its monomeric (“closed” or autoinhibited) state, an acidic C-terminal helix binds the basic N-terminal interface responsible for membrane binding and oligomerization. In experimental settings, deletion of the acidic C-terminal helix of multiple CHMPs results in the formation of an insoluble membrane-bound polymer that disrupts MVB biogenesis and retrovirus budding [25, 34, 36, 37]. The C-terminus of CHMPs also contains protein-binding sites for upstream factors of the ESCRT machinery, such as Eap20 and AIP1/Alix. This raises the possibility that protein binding displaces the autoinhibitory C-terminal helix to promote ESCRT-III array formation. Interestingly, this “open” conformational state exposes an MIT-interacting motif that allows ESCRT-III to recruit Vps4. Though ESCRT-I and -II may function in sorting cargo to MVBs, ESCRT-III polymerization may function in vesicle formation and membrane scission. A recent study using quick-freeze deep-etch electron microscopy demonstrated that overexpressed CHMP4 polymerized on the plasma membrane and endosomes as curved filaments assembled into a circular array [38]. When coexpressed with Vps4B_E235Q_, the CHMP4 polymers formed buds and tubules in which the membrane folded away from the cytoplasm [38]. Similarly, C-terminally deleted fragments of CHMP2A and CHMP3 coassembled into helical tubular structures in vitro, in which the membrane-binding site was exposed on the outside surface and the Vps4-binding site was enclosed within the hollow tube [39, 40]. When the CHMP2A/3 tubules were assembled in vitro with Vps4B, Vps4B was oligomerized on the inside of the tubes. In the presence of ATP, Vps4B catalyzed the disassembly of these structures [39, 40]. In the context of retrovirus budding, Gag oligomerization may be sufficient for membrane deformation. ESCRT-III may facilitate Vps4 recruitment to the site of assembly in order to provide the energy required for membrane scission. Defining the ESCRT-III subunits required for retrovirus budding is an active area of research. However, assessing the role of ESCRT-III subunits has proven difficult thus far. Dominant-negative forms of various ESCRT-III proteins, in which the C-terminal autoinhibitory domain is deleted, display potent inhibition of HIV-1 and ASLV Gag release [34, 36]. Whether dominant-negative interference by ESCRT-III fragments reflects a direct interference of the retrovirus budding complex or an indirect sequestration of necessary cofactors is not known. Similarly, siRNA-mediated knockdown of various ESCRT-III proteins has yielded limited insights to date [34, 35]. Whether siRNAs fail to sufficiently deplete endogenous proteins or if ESCRT-III contains redundant mechanisms is unclear. 

## 3. Positive Sorting Signals

Current research recognizes that monoubiquitin and charged lipids provide critical sorting signals for the recruitment and activation of ESCRT complexes. Similarly, retroviruses rely on these sorting signals to complete the budding process.

Ubiquitin, a 76-amino acid protein, regulates several cellular processes ranging from proteasome-mediated degradation to DNA repair to protein transport. Posttranslational attachment of a single ubiquitin moiety (i.e., monoubiquitin) serves as a signal for transmembrane protein internalization and sorting through the endocytic pathway. Several endocytic proteins contain ubiquitin-binding domains to recognize the monoubiquitin sorting signal [23]. The first endocytic protein to sense ubiquitinated cargo during MVB biogenesis is the class E Vps protein, Hrs, which forms part of the Hrs-Stam complex. The Hrs-Stam complex localizes to early endosomes (through the FYVE domain of Hrs) to direct ubiquitinated cargo to the limiting membrane of late endosomes. Hrs also interacts with Tsg101, and recruits ESCRT-I to the endosomal membrane. ESCRT-I initiates the recruitment of downstream components of the MVB pathway, such as ESCRT-II, AIP1/Alix, or ESCRT-III, to the limiting endosomal membrane. Importantly, both ESCRT-I and -II contain ubiquitin-binding domains that may allow for sorting and concentration of ubiquitinated cargo at the site of vesicle formation. Though ESCRT-III proteins do not bind ubiquitin, several bind to deubiquitinating enzymes, such as AMSH and UBPY, to remove ubiquitin from cargo proteins prior to membrane scission [41].

In addition to ubiquitin, lipid sorting also plays an important role in establishing appropriate platforms for MVB biogenesis. Unlike the limiting endosomal membrane, vesicle membranes must be susceptible to the hydrolytic environment for degradation. Several observations suggest that the lipid phosphatidylinositol 3-phosphate (PI(3)P) provides critical functions for vesiculation [42]. PI(3)P localizes to the cytoplasmic leaflet of early endosomes and internal vesicles of MVBs. Several proteins along the endocytic pathway, such as Hrs, Eap45 (ESCRT-II), and Chmp4 (ESCRT-III), contain PI(3)P-binding domains for localization to sites of MVB biogenesis [23]. Inhibitors of PI 3-kinases, the enzymes that phosphorylate phosphatidylinositols to produce PI(3)P, prevent vesiculation and cause cargo proteins, such as EGFR, to remain trapped on the limiting membrane of MVBs [43]. In addition to PI(3)P, other lipids, including cholesterol and lysobisphosphatidic acid (LBPA), function in the subdomain organization and effector protein recruitment [23, 42].

**Ubiquitin**

The first indications that ubiquitin might play a role in retrovirus replication came from studies in which purified ASLV particles were found to contain a significantly greater concentration of unconjugated ubiquitin than that present in the cytosol [44]. The fact that the relative amounts of other low molecular weight host protein were not increased suggested that ubiquitin was selectively incorporated into virions [44]. Subsequently, studies with HIV-1, simian immunodeficiency virus (SIV), and MLV Gag verified that ~2–5% of Gag in VLPs were monoubiquitinated [45]. Additionally, the ubiquitin moieties were covalently attached to the L domain-encoding p6 and p12 peptides of HIV-1/SIV and MLV Gag, respectively [45]. This finding correlates with several observations that Gag is ubiquitinated in an L domain-dependent manner. Several lines of evidence point to a role for ubiquitin in the budding process: (1) depleting soluble ubiquitin by treating cells with proteasome inhibitors causes a late budding defect for most retroviruses [46, 47]. Fusing ubiquitin to the C-terminus of ASLV Gag rescues budding in the presence of inhibitor treatment [48]; (2) substitutions of lysine residues in close proximity to the L domain of HIV-1, ASLV, and HTLV-1 Gag inhibit VLP release [49–51]; (3) as mentioned previously, overexpressing catalytically inactive Nedd4-like E3 ligases inhibit budding of PPxY-dependent retroviruses [22, 29]; (4) overexpressing ubiquitin bearing mutations in hydrophobic residues that regulate endocytic signaling also inhibit HIV-1 Gag release [52].

Despite the known requirement for ubiquitin in retroviral budding pathways, the mechanistic function of ubiquitin remains unsolved. One model posits that ubiquitination of Gag allows for increased affinity between Gag and ESCRT components required for budding, specifically ESCRT-I and -II complexes. For example, the ubiquitin-binding domain of ESCRT-I resides within the N-terminal UEV domain of Tsg101, the host factor that binds the PTAP motif of HIV-1 Gag. Ubiquitination of the p6 region of HIV-1 Gag actually increases its binding affinity to Tsg101 [53]. Conversely, deleting the Ub-binding pocket within the UEV domain of Tsg101 potently inhibits the release of HIV-1 Gag [54]. Further evidence for the role of ubiquitin in facilitating assembly of Gag/ESCRT complexes comes from observations that fusion of ESCRT-I and -II proteins to the C-terminus of ASLV Gag/Δp2b not only rescued budding of Gag, but also restored ubiquitin modification that was lost upon the deletion of the L domain (see Figure 1) [34]. A fraction of the Gag-ESCRT chimeras incorporated into VLPs was monoubiquitinated [33, 34]. Interestingly, fusion of the ESCRT-III subunit, Chmp6, failed to restore ubiquitination of the Gag/Δp2b-ESCRT chimera despite its low level of budding [34]. These findings indicate that (i) efficient budding of VLPs correlates with ubiquitin modification, and (ii) ubiquitination also coincides with the utilization of ESCRT-I and/or -II complexes during budding. This latter point is consistent with the fact that only the ESCRT-I and -II complexes contain known ubiquitin-binding elements capable of recognizing this sorting signal. In fact, a recent study showed that fusing ubiquitin to the C-terminus of the L domain-deficient EIAV Gag/ΔYPDL rescued particle production in a Tsg101-dependent budding pathway [55]. Budding rescue relied on the surface-exposed hydrophobic residues on ubiquitin that mediate the interaction with ubiquitin-binding domains found in Tsg101 and, possibly, AIP1/Alix [55].

Other observations offer alternative roles of ubiquitin in retrovirus budding. For example, ubiquitin fusion to the C-terminus of EIAV Gag/ΔYPDL sensitized VLP release to proteasome inhibitors [55]. This suggests that ubiquitination of factors other than Gag may be necessary for particle release. Additionally, the Gag protein of the prototypic foamy virus (PFV), which encodes a PSAP L domain, contains a single lysine residue that bears no requirement for VLP release [56]. Replacing the PSAP motif of PFV Gag with the PPPY motif of MLV Gag renders this chimeric PFV Gag-PY construct dependent on Nedd4-like E3 ligases for budding. Yet, budding remains unaffected when substituting the single ubiquitin acceptor site from this PFV Gag-PY construct [56]. These findings suggest that ubiquitination of Gag is not necessary for particle release. It should be noted, however, that PFV exhibits unusual assembly properties since capsids assemble in the cytosol and VLPs are only released when Gag is coexpressed with Env. The PFV Gag constructs described by Zhadina et al. appended artificial membrane-targeting domains to the N-terminus of PFV Gag to eliminate the requirement for Env coexpression in VLP production.

**Lipids**

The current model proposes that the M domain, located within the matrix regions of Gag, mediates plasma membrane binding for assembly and budding. For most retroviruses, the M domain signal consists of a series of conserved basic residues and an N-terminal acyl group, typically myristate, covalently attached upon translation. Several cellular proteins are known to bind membranes through a myristyl switch mechanism, in which a conformational change triggers the exposure of myristate to promote membrane association [57, 58]. Strong evidence supports the view that myristylated Gag interacts with membranes through a similar mechanism [59–61]. For example, monomeric Gag of HIV-1, which binds membranes poorly, sequesters the N-terminal myristate moiety within the MA globular domain. Gag oligomerization in the cytosol coincides with myristate exposure, drastically increasing membrane affinity. The cluster of basic amino acids in MA appears to impart specificity to membrane binding. Substitution of these basic residues impedes particle release by misdirecting Gag assembly toward intracellular membrane platforms [62]. Possibly, the basic domain within MA interacts with host cell factors to determine the site of assembly and budding. Cells contain multiple variations of phosphatidylinositols (PI), classified by the number and position of phosphate groups attached to the inositol ring. Different PIs localize to different subcellular compartments to direct proteins to specific sites of action. PI(4,5P_2_, along with PI(3,4,5)P_3_, accumulates on the cytoplasmic leaflet of the plasma membrane. Recent findings point to the lipid phosphatidylinositol (4,5)-bisphosphate (PI(4,5)P_2_) in regulating HIV-1 Gag trafficking to the plasma membrane [63]. Depleting PI(4,5)P_2_ in HeLa cells by overexpressing PI-5-phosphatase IV (5-ptase IV) reduced HIV-1 Gag budding and targeted Gag to CD63-positive late endosomes [64]. A recently described NMR structure of the myristylated HIV-1 MA protein demonstrates that the 2 fatty acid chain of PI(4,5)P_2_ occupies a hydrophobic cavity within MA, and that the negatively charged phosphate groups on the inositol ring interact with basic residues in MA.Similar findings were reported for the MA protein of the related retrovirus, EIAV [65]. Critically, PI(4,5)P_2_ binding changes the conformation of Gag such that the myristyl group becomes exposed, thereby coupling plasma membrane binding to assembly [66].

The transport signal for ASLV Gag may differ from HIV-1 Gag as suggested by the disparate localization patterns observed for fluorescent-tagged ASLV Gag-GFP and HIV-1 Gag-RFP when coexpressed in mammalian cells. [67]. Unlike the myristyl switch of HIV-1, ASLV MA relies only on the patch of basic residues for membrane binding. However, recent observations also suggest that ASLV Gag associates with specific membrane components to determine the site of assembly and budding. Though phosphatidylethanolamine (PE) is a major component of the plasma membrane, the lipid analog N-Rh-PE is a lipid marker for endocytic vesicles because it forms small molecular clusters when present in membranes [57]. Molecular aggregation has been proposed to function as an additional positive sorting signal for lysosomal targeting since membrane components may be targeted to the lysosome when induced to aggregate. The presence of a rhodamine fluorophore on the head group of N-Rh-PE enables the visualization of endosome-derived membranes by confocal microscopy [68, 69]. When VLPs are purified from COS cells treated with N-Rh-PE, ASLV Gag-GFP particles contain the lipid analog in their envelope (see Figure 1) [31]. Interestingly, HIV-1 Gag VLPs fail to incorporate N-Rh-PE [31]. These findings suggest that ASLV Gag and HIV-1 Gag bud through different membrane regions, with ASLV Gag passing through an endosome-derived membrane at some point during the assembly process (see Figure 1). Furthermore, ASLV Gag appears to associate with N-Rh-PE-positive membranes in an L domain-dependent manner. Unlike VLPs assembled from WT Gag, the VLPs assembled from ASLV Gag/Δp2b do not incorporate N-Rh-PE or the tetraspanin protein CD63 (a protein marker of the LE/MVB compartment) [31]. The finding that the L domain may contribute to membrane targeting of Gag was surprising because L domains are not typically thought to play a role in transport. Unlike the C-terminal p6 or p9 regions of HIV-1 or EIAV Gag, respectively, factors recruited by the N-terminal p2b region of ASLV may cooperate with the M domain to form the transport signal. Possibly, the C2 transport domain of Nedd4-like proteins may function in concert with the M domain signal. Alternatively, ASLV may utilize the membrane-binding activity of the ESCRT complexes to associate with N-Rh-PE-positive membranes. In support of this view, VLPs assembled from the chimeric Gag construct, ASLV Gag/Δp2b-Eap20, incorporated the N-Rh-PE tracer into the envelope (see Figure 1) [31]. When ASLV Gag adopts a Tsg101-dependent budding pathway, as in the case of the Gag-ESCRT-I fusions (ASLV Gag/Δp2b-Tsg101 or −Vps37C), VLPs failed to incorporate N-Rh-PE (see Figure 1) [31]. This directly demonstrates that factors recruited by different L domains confer different budding properties on Gag. Similar to the exchange of L domains, alteration of the proteins that form the budding complex (i.e., substituting Tsg101/Vps37C for Nedd4-like E3s) redirects Gag toward an alternate budding pathway.

## 4. Conclusion

Substantial progress has been made in recent years to elucidate the role of different ESCRT complexes in retrovirus budding and to understand the role that monoubuiqitination of Gag plays in assembling the budding complexes. However, fundamental questions still remain. For example, after structural and biochemical data allowed for better understanding of the ESCRT-I and ESCRT-II complexes, the focus now turns to ESCRT-III. How do ESCRT-III subunits oligomerize on membranes? What signals regulate this oligomerization? Which ESCRT-III proteins are required for retrovirus budding? Novel strategies may be required to answer this latter question, as dominant-negative interference and siRNA-mediated knockdown of various CHMPs appear inadequate. Furthermore, additional questions remain about the role of ubiquitin in retrovirus budding. Is monoubiquitin modification of Gag a necessary aspect of the budding pathway or merely a byproduct of the ESCRT complexes associating with ubiquitination apparatus? The requirement for monoubiquitin may depend on which retroviruses recruit host factors capable of recognizing the ubiquitin signal, and may not be a universal requirement for budding. One unifying theme seems clear: retroviruses may utilize parallel budding pathways by co-opting different components of the ESCRT machinery to reach the same end point, that is, Vps4-dependent release of particles from the plasma membrane.
